# Effect of High-Temperature Stress on Fatty Acid Composition and Undecylprodiginine Biosynthesis in *Streptomyces coelicolor* M511

**DOI:** 10.3390/microorganisms13112520

**Published:** 2025-11-01

**Authors:** Youngjong Han, Yujun Park, Kyudong Han, SangJoon Mo

**Affiliations:** 1 Department of Microbiology, College of Science & Technology, Dankook University, Cheonan 31116, Republic of Korea; hanyj0918@gmail.com (Y.H.); qkrwodbwns1109@gmail.com (Y.P.); 2 Smart Animal Bio Institute, Dankook University, Cheonan 31116, Republic of Korea; 3 Department of Human, Microbiome Research HuNbiome Co., Ltd., R&D Center, Seoul 08507, Republic of Korea

**Keywords:** undecylprodiginine, branched-alkyl prodiginine, FabH, RedP, heat stress

## Abstract

Actinomycetes are a representative group of bacteria that inhabit soil; in particular, *Streptomyces coelicolor* M511 produces actinorhodin and undecylprodiginine. Among them, undecylprodiginine has antibiotic and immunosuppression activity and is a secondary metabolite with high potential applications in biotechnological and pharmaceutical fields. High temperature stress (37 °C) reduced the biosynthesis of undecylprodiginine and induced specific branched chain alkylprodiginine derivatives, compared with the optimal growth temperature (30 °C). Also, the stress stimulated the synthesis of straight-chain FA for enhancing membrane rigidity. The inhibition of undecylprodiginine biosynthesis under high temperature stress seems to be induced by the heat sensitivity of the RedP enzyme, and this inhibition is compensated by FAS FabH. Since FabH, a homologue of RedP, has a broader substrate specificity, it leads to the production of methylundecylprodiginine and methyldodecylprodiginine. The external addition of isoleucine (as well as that of leucine and valine to a far lesser extent) enhances the synthesis of these derivatives since isoleucine catabolism generates precursors used for the biosynthesis of these compounds. These findings reveal temperature-dependent changes in precursor utilization and prodiginine diversity, providing insights into metabolic plasticity and strategies establishing a foundation for secondary metabolite derivatives engineering strategies through precursor supplementation or temperature regulation.

## 1. Introduction

Actinomycetes are a representative group of bacteria that primarily inhabit soil. In particular, *Streptomyces* is the largest genus of actinomycetes, characterized by complex growth structures and the production of various useful natural substances [1]. They are classified as Gram-positive bacteria that inhabit various environments and have cell walls with multiple layers formed by teichoic acid [2]. *Streptomyces* are taxonomically grouped as bacteria but exhibit a unique multicellular developmental pattern, forming a widely branched substrate mycelium and aerial hyphae [3]. One of the most notable features of the *Streptomyces* genus is its ability to produce a wide range of antibiotics and biologically active secondary metabolites, including immunosuppressants, antihypertensive agents, and antiviral compounds [4,5].

In particular, *Streptomyces coelicolor* A3 (2) has been commonly used as a model strain for studies on antibiotic biosynthesis regulation among various bacterial strains. This strain produces four structurally distinct antibiotics: actinorhodin, undecylprodiginine, a calcium-dependent antibiotic, and methylenomycin [6,7]. Among these, undecylprodiginine is a substance that exhibits various biological activities, including antibacterial, antifungal, immunosuppressive, and anticancer properties, and has been continuously studied due to its high potential for biological applications [8,9]. In this study, the *S. coelicolor* M511 strain was used to investigate undecylprodiginine. In the strain M511, the gene encoding *actII-ORF4,* the specific regulator of the ACT pathway, is deleted. Thus, this strain serves as a useful system to investigate undecylprodiginine production, since actinorhodin is not synthesized and RED accumulation is markedly enhanced [10].

Fatty acid synthases (FAS) could be classified into two types, I and II [11]. Type I FAS are multifunctional protein complexes covalently linked to the acyl carrier protein (ACP) and are found only in a few microorganisms, such as Mycobacterium and Corynebacterium [12]. In contrast, type II FAS are composed of individual proteins that catalyze specific steps and are present in most bacteria, including *Streptomyces* [13].

Most type II FAS are produced from branched-chain starter units such as isobutyryl-CoA, isovaleryl-CoA, and anteiso-valeryl-CoA. These pathways ultimately produce odd- or even-chain fatty acids with a methyl branch at the terminal position. Iso- and anteiso-fatty acids, branched-chain fatty acids (BCFAs), have a methyl group substituted on the second or third carbon from the terminal end. These features of structure derived from amino acid precursors (e.g., leucine, isoleucine, and valine) can provide membrane fluidity regulation functions in response to temperature stress [14]. However, a few FAS of type II can synthesize straight-chain fatty acids (SCFAs) from starter units such as acetyl-CoA or propionyl-CoA [15,16]. The characteristic of FabH, a major enzyme involved in the initial synthesis of FAS II, which shows no difference in substrate discrimination between straight and branched substrates but exhibits differences in preference, has also been reported in other studies.

The 3-ketoacyl-ACP synthase III, FabH, is the key enzyme that initiates fatty acid synthesis in the type II FAS pathway of *Streptomyces coelicolor*. FabH catalyzes the decarboxylative condensation of acyl-CoA and malonyl-ACP to yield 3-ketoacyl-ACP. Subsequently, FabG reduces 3-ketoacyl-ACP to 3-hydroxyacyl-ACP, which is dehydrated by FabA to generate enoyl-ACP. In the next step, FabI (InhA) reduces enoyl-ACP to saturated fatty acids, thereby completing one elongation cycle, while FabF mediates iterative chain extension [17]. FabH enzymes are generally able to utilize both straight-chain and branched-chain acyl-CoA substrates [18].

**Figure 1:**
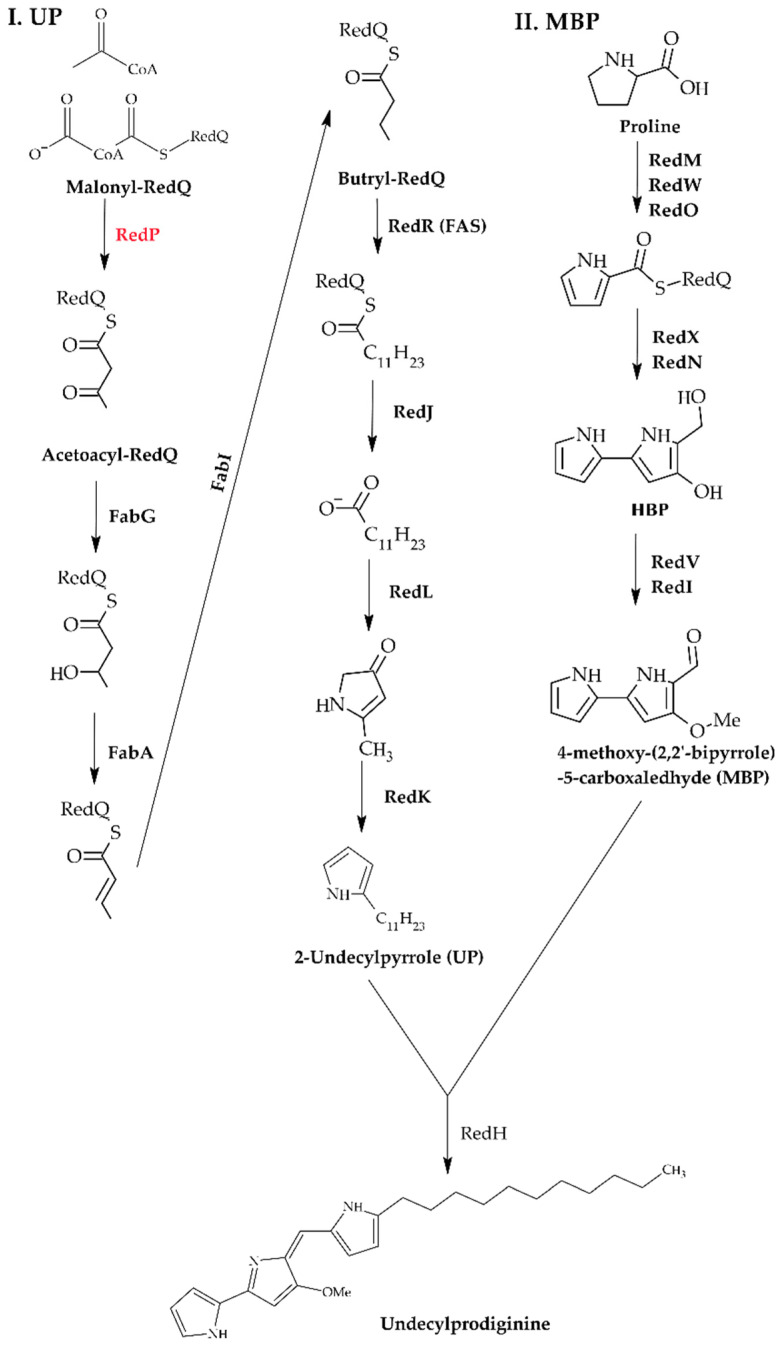
The biosynthesis process of undecylprodiginine in *S. coelicolor* strains. I. Biosynthetic steps of undecylpyrrole (UP): Acyl-CoA reacts with malonyl-RedQ via RedP to initiate synthesis, which then proceeds through enzymes related to fatty acid synthesis, such as FabG and FabA. Finally, UP is formed through RedR, RedJ, RedL, and RedK. II. Biosynthetic steps of 4-methoxy-(2,2′-bipyrrole)-5-carbaldehyde (MBP): Using a proline-derived precursor as the starter unit, MBP is formed through a series of reactions catalyzed by enzymes such as RedM, RedW, and RedO. UP and MBP, formed in the two pathways, are bound by RedH and ultimately synthesize undecylprodiginine.

In *S. coelicolor*, RedP, a homolog of the type II FAS FabH, is also involved in the biosynthesis of 2-undecylpyrrole (UP), a precursor of undecylprodiginine. The formation of UP begins with RedP that catalyzes the condensation of acetyl-CoA and malonyl-RedQ to form a 3-ketoacyl intermediate, which is then reduced by type II FAS enzymes (FabG, FabA, FabI, FabH) to produce butyryl-RedQ [9]. RedR then catalyzes four iterative elongation cycles using malonyl-RedQ, with each 3-ketoacyl intermediate undergoing sequential processing. RedJ hydrolyzes dodecanoyl-RedQ to release dodecanoic acid, which RedL then converts into 4-keto-2-undecylpyrroline. RedJ exhibits marked specificity toward acyl substrates of C_10_ or longer, enabling it to discriminate acyl-RedQ from other acyl-ACP [19,20]. At last, RedK catalyzes the reduction and dehydration of 4-keto-2-pyrroline to generate UP. MBP, 4-methoxy-(2,2′-bipyrrole)-5-carboxaldehyde, is synthesized via a pathway distinct from that of UP (Figure 1). In the first step of MBP biosynthesis, RedM, RedO, and RedW catalyze the formation of a pyrrole ring from proline. In the subsequent step, RedX and RedN catalyze the decarboxylation and condensation reaction of L-serine and malonyl-CoA, resulting in the formation of 4-hydroxy-2,2′-bipyrrole-5-methanol (HBP) [21]. The UP and MBP produced in these two separate pathways are then condensed by RedH, ultimately yielding the tripyrrole pigment undecylprodiginine (Figure 1) [9].

Previous studies have indicated that RedP and FabH are homologues, but they differ in substrate preference. RedP catalyzes only the condensation of acetyl-CoA with malonyl-RedQ. In contrast, FabH can catalyze condensations of diverse acyl-CoA substrates (such as acetyl-CoA and isobutyryl-CoA) with various ACPs, including malonyl-RedQ or malonyl-FabC. However, its highest activity occurs when isobutyryl-CoA is combined with malonyl-FabC. This substrate-level specificity enables FabH to distinguish fatty acid synthesis from undecylprodiginine biosynthesis [22,23]. It has been reported that in strain M511 lacking the RedP enzyme (mutant strain SJM1, *ΔredP*), undecylprodiginine production decreased by approximately 80%, and that simultaneously, a new form of branched-chain alkylprodiginines was produced at low levels. Interestingly, the overexpression of FabH led to a partial recovery of the undecylprodiginine production and enhanced that of branched-chain alkylprodiginines. These findings indicate that undecylprodiginine biosynthesis can be initiated by FabH, a fatty acid synthase enzyme [24]. Undecylprodiginine and fatty acids are synthesized through distinct metabolic pathways; however, the shared use of enzymes such as FabH, FabG, and FabI indicates that these pathways are interconnected. In this study, we aimed to investigate the impact of environmental stress associated with high temperature in *S. coelicolor*, as this topic has not been previously addressed. Specifically, we examined the impact of heat stress on the biosynthesis of undecylprodiginine and on that of fatty acids. Heat stress has been shown to broadly influence bacterial metabolism, including growth retardation, induction of heat shock proteins (HSPs), and alterations in fatty acid composition [25,26]. For instance, when *Escherichia coli* O157:H7 was cultured at 46 °C, the proportion of saturated fatty acid (C_16:0_) increased while that of unsaturated fatty acid (C_18:1_) decreased. These changes resulted in increased cell membrane rigidity, leading to heat resistance [27]. These findings demonstrate that bacteria adapt to high-temperature stress by adjusting their membrane lipid composition, partly through the regulation of FAS-related enzyme expression and activity [28].

Despite numerous studies about the fatty acids and metabolites of the *S. coelicolor* strain, research on the effects of changes in the strain under high-temperature stress conditions is still limited. Therefore, this study aimed to investigate how changes in the fatty acid synthesis pathway induced by heat stress impact the biosynthesis of undecylprodiginine.

## 2. Materials and Methods

### 2.1. Strain Information and Culture Conditions

*S. coelicolor* M511 (SCP1-, SCP2-, act-, (∆*actII*-*ORF4*)) was provided by Dr. Greg Challis (Warwick University, Coventry, UK). M511 spore solution (10 µL) was inoculated into a baffled flask containing 50 mL of R2YE medium and incubated at 30 °C for 28 h. Then, 50 mg of wet cells were quantified and inoculated into 50 mL of fresh R2YE medium. Subsequently, culture was conducted for four days in a shaking incubator at 30 °C. In the case of heat stress, culture was conducted at 37 °C after reinoculation. After the culture was completed, the mixture was centrifuged at 4200 rpm for 10 min, and only the cell pellet was obtained using a paper filter (Hyundai micro, Anseong, Republic of Korea).

### 2.2. Leucine, Isoleucine, Valine, and Chemical Feeding Studies in High Temperature Stress Conditions

We aimed to verify whether the addition of DL-valine (≥99%, Junsei, Tokyo, Japan), L-isoleucine (≥99%, Samchun, Pyeongtaek, Republic of Korea), and L-leucine (≥98%, Sigma-Aldrich, St. Louis, MO, USA), which are amino acids that provide precursors for the biosynthesis of BCFAs in *Streptomyces* strains, affects the production of undecylprodiginine. The culture of strain was performed as described above. After 24 h of incubation at 37 °C, leucine, isoleucine, and valine were added to the 50 mL of medium by calculating the respective weights to adjust a final concentration of 10 mM.

### 2.3. HPLC Analysis of Prodiginine Extract from M511 Strain

Before high-performance liquid chromatography (HPLC) analysis, one of the chromatographic analysis, we modified the extraction method described in a previous study [29]. To extract undecylprodiginine from the cell pellet, 100 mg of cells were treated with 5 mL of methanol (in 10% HCl, 10% *v*/*v*). The mixture was then incubated at room temperature for three days to allow complete extraction of the substance. After incubation, mixtures were centrifuged at 4200 rpm for 5 min to settle the cell pellet. Then, the upper layer of methanol containing undecylprodiginine was evaporated under reduced pressure using a Laborota 4000 Rotary Evaporator (Heidolph, Schwabach, Germany).

The concentration of undecylprodiginine in all extracts was measured at a wavelength of 530 nm (ϵ530 = 100, 500 M^−1^ cm^−1^) using a UV-vis spectrophotometer 2600i (Shimadzu, Kyoto, Japan). All data were performed independently in triplicate and analyzed using GraphPad Prism version 10.6.1 (Boston, MA, USA) for a one-way analysis of variance (ANOVA). Homogeneity of variances was verified using the Brown–Forsythe test and Bartlett’s test.

HPLC analysis of undecylprodiginine was performed using an HPLC 1290 series (Agilent, Santa Clara, CA, USA) and a Discovery C_18_ column (25 cm × 4.66 mm, 5 μm particle size, Supleco, Bellefonte, PA, USA). The injection volume was 10 μL, and the flow rate was set to 0.5 mL/min. The detailed solution gradient was performed as described in the supplementary data (Table S1) [24].

### 2.4. Verification of Undecylprodiginine in the Spectrum Using LC-QTOF-MS

For the analysis of undecylprodiginine, methyl-undecylprodiginine, and methyl-dodecylprodiginine, we employed liquid chromatography-mass spectrometry (LC-MS) analysis, a combination of chromatographic and mass spectrometric techniques. We extracted a metabolite from the M511 strain. Moreover, analysis was performed using a quadrupole time-of-flight mass spectrometer (QTOF-MS) 6546 series (Agilent, Santa Clara, CA, USA) at the Center for Biomedical Engineering Core Facility (Dankook University, Cheonan, Republic of Korea). LC-MS analysis conditions were modified from those reported previously [30]. For the analysis of the hydrophobic compound undecylprodiginine, a ZORBAX Eclipse Plus C_18_ column (Rapid Resolution HD 2.1 × 150 mm, 1.8 μm, Agilent, Santa Clara, CA, USA) was used. The mobile phases consisted of water (HPLC grade; J.T. Baker, Avantor, Allentown, PA, USA) containing 0.2% formic acid and acetonitrile (HPLC grade; J.T. Baker, Avantor, Allentown, PA, USA) containing 0.2% formic acid. The injection volume was 5 μL, and the flow rate was set to 0.3 mL/min. The gradient changes in the mobile phase and mass analysis were performed as outlined in the tables (Tables S2 and S3).

### 2.5. Confirmation of Fatty Acid Profiling in Each Culture Group Using GC-MS

To extract fatty acids from the M511 strain, 3 mL of methanol (in 10% HCl, 10% *v*/*v*) was added to 100 mg of cell pellet, and the mixture was heated at 80 °C for 1 h to break down the cell wall and methylate the cells. After heating, the sample was cooled to room temperature, and 1 mL of 0.75% NaCl and 3 mL of hexane were added to extract only fatty acid methyl ester. The upper layer containing fatty acids dissolved in hexane was recovered by centrifugation and concentrated under reduced pressure. The concentrated sample was redissolved in 1 mL of hexane and used for analysis [31].

The gas chromatographic mass spectrometry (GC-MS) analysis conditions for fatty acid methyl ester (FAME) samples were modified according to the fatty acid analysis report [32]. Helium (purity > 99.999%) was used as the carrier gas, with a flow rate maintained at 1.0 mL/min. Detailed conditions can be found in the supplementary data (Table S4). Fatty acid profiling and ratio under each condition were conducted by using Unknown Analysis and Qualitative Analysis 10.0 software (Agilent, Santa Clara, CA, USA). The statistical significance of the fatty acid ratio under each condition was tested using one-way ANOVA.

## 3. Results

### 3.1. Heat Stress During the Growth of the M511 Strain Reduces the Production of Undecylprodiginine and Generates Branched-Chain Alkylprodiginines

**Figure 2:**
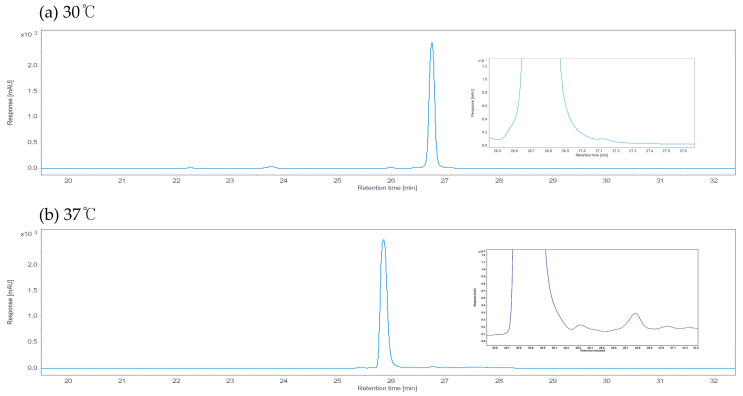
HPLC chromatograms of undecylprodiginine from M511 strains cultured at 30 °C and 37 °C conditions. The *x*-axis is retention time (RT), and the *y*-axis is response [mAU]. Undecylprodiginine was extracted from each culture sample using an organic solvent, and the peaks were confirmed by HPLC analysis. (**a**) At 30 °C, only one peak was detected. (**b**) At 37 °C, three distinct peaks were detected.

**Figure 3:**
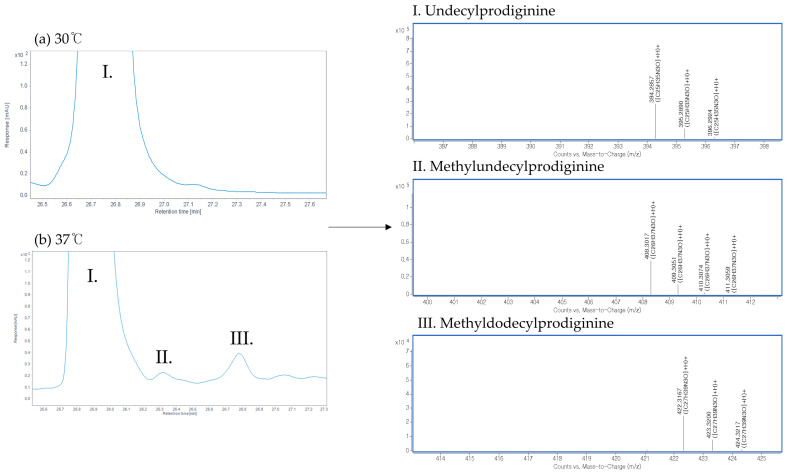
LC-MS spectra of undecylprodiginine and branched-chain alkylprodiginine from the M511 strains cultured at 30 °C and 37 °C. The *x*-axis is mass to charge (*m*/*z*+), and the *y*-axis is counts. (**I**) Undecylprodiginine with an *m*/*z* of 394.2857, (**II**) Methylundecylprodiginine with an *m*/*z* of 408.3017, and (**III**) Methyldodecylprodiginine with an *m*/*z* of 422.3167. LC-MS analysis of prodiginine extracts used in HPLC analysis was performed. (**a**) At 30 °C, only the spectrum of undecylprodiginine was observed, while (**b**) at 37 °C, the spectrum of branched alkyl prodiginine, including undecylprodiginine, was detected.

**Figure 4:**
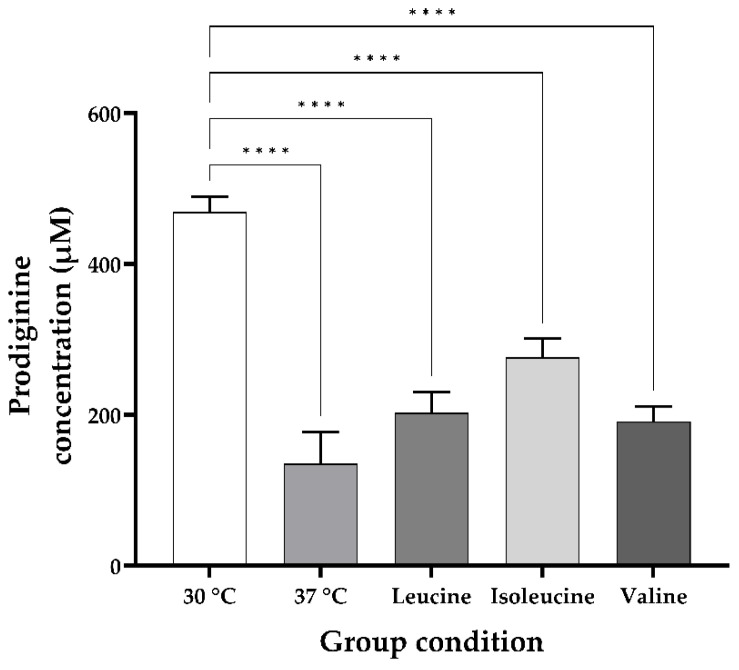
Total prodiginine concentration of the M511 strains in each condition. The concentrations of prodiginine were 468.9 (±20.83), 135.3 (±42.18), 202.7 (±27.4), 275.9 (±24.77), and 191 (±20.2) μM, respectively. The *p* values were calculated by comparing each treatment group with the 30 °C group. **** *p* < 0.0001.

To evaluate changes in the undecylprodiginine production pattern of *S. coelicolor* M511 under heat stress, cultures were grown at 30 °C and 37 °C, respectively. Undecylprodiginine was extracted from each sample and analyzed by HPLC. The main peak, estimated to be undecylprodiginine, was detected at approximately 26 min (±1) on the chromatogram in both conditions (Figure 2). As shown in Figure 2a, only a unique undecylprodiginine peak was observed in the M511 strain cultured at 30 °C. In contrast, under the 37 °C culture condition shown in Figure 2b, in addition to the main peak of undecylprodiginine, minor peaks were detected at far lower concentrations at adjacent positions. This result suggested that chemical derivatives of undecylprodiginine were generated under heat stress conditions. These HPLC chromatogram patterns were similar to those of the *S. coelicolor* (*ΔredP*) mutant strain SJM1, as reported in previous studies [24]. Accordingly, LC-MS analysis was performed to confirm whether the peaks detected at 37 °C in this experiment matched those reported in previous studies as branched-chain alkylprodiginines. Undecylprodiginine was detected at a retention time of 20.3–20.4 min by LC-MS in the extract of the M511 strain cultured at 30 °C. The *m*/*z* value of the main spectrum was 394.2857 in the [M+H]+ state, which matched the ion signal corresponding to the molecular formula C_25_H_35_N_3_O of undecylprodiginine (Figure 3). On the other hand, under heat stress conditions at 37 °C, LC-MS analysis of the M511 strain revealed the detection of additional substances at 21.0 ± 0.4 min and 22.0 ± 0.4 min, with *m*/*z* values of 408.3017 and 422.3167, respectively. These values matched the spectral data reported for methylundecylprodiginine and methyldodecylprodiginine [24]. To determine the concentration of undecylprodiginine in M511 under the two temperature conditions, we performed absorbance quantification followed by calculation of the molar concentration. The total prodiginine concentration was 468.9 (±20.83) μM at 30 °C but decreased to 135.3 (±42.18) μM at 37 °C, representing a reduction of approximately 71.15% (a 3.46-fold reduction) (Figure 4). Statistical analyses of prodiginine concentration were performed as described in Supplementary Tables S5 and S6. These results indicate that heat stress leads to a significant reduction in prodiginine biosynthesis while promoting the formation of new branched-chain alkylprodiginine derivatives.

### 3.2. Changes in the Undecylprodiginine Production Pattern When BCAA Is Added in a Heat Stress Environment

**Figure 5:**
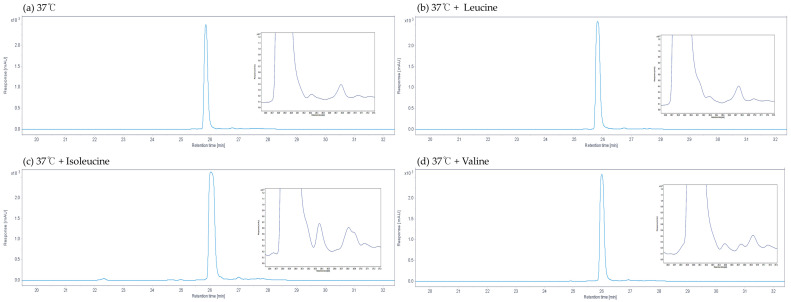
HPLC chromatograms of undecylprodiginine from the M511 strains cultured at 37 °C with BCAA addition. The *x*-axis is retention time (RT), and the *y*-axis is response [mAU]. Undecylprodiginine was extracted in the same manner as in the previous process and analyzed by HPLC. (**a**) At 37 °C, (**b**) 37 °C with leucine added, (**c**) 37 °C with isoleucine added, and (**d**) 37 °C with valine added. All BCAA peaks were detected at 37 °C under the same conditions.

**Figure 6:**
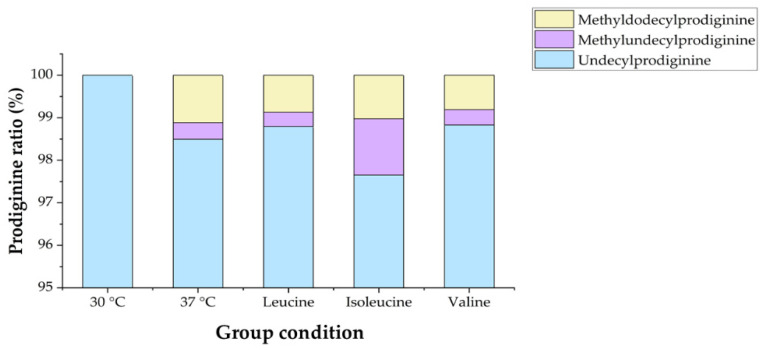
Each prodiginine ratio of M511 strains in each condition. The analysis was performed without an internal standard, and the relative proportions of each prodiginine derivative were calculated based on peak areas obtained from the HPLC chromatogram. Peak areas were determined using the peak integration function in OpenLab CDS.

The impact of adding branched-chain amino acids (BCAAs) to the growth medium on undecylprodiginine biosynthesis was examined. The bacteria were cultured under conditions of heat stress at 37 °C, and the BCAAs leucine, isoleucine, and valine were added during the cultivation process. HPLC analysis was performed under the same conditions. The HPLC chromatograms revealed one main peak and two minor peaks, similar to the control grown at 37 °C without BCAA addition (Figure 5). LC-MS analysis was then performed to verify the nature of the detected compounds, and spectra with *m*/*z* values of 408.3017 and 422.3167, identical to those detected at 37 °C in the BCAA-added group, were confirmed as methylundecylprodiginine and methyl-dodecylprodiginine. Furthermore, Figure 4 indicated that when leucine, isoleucine, and valine were added individually at 37 °C, the prodiginine concentration increased from the control with no BCAA addition, 135.3, to 202.7 (±27.4), 275.9 (±24.77), and 191 (±20.2) μM, respectively. Among these, isoleucine resulted in higher prodiginine production than leucine and valine (2.04-fold compared with the 37 °C no-supplementation group). Isoleucine supplementation also exerted a greater stimulatory effect than leucine or valine on the biosynthesis of the branched-chain derivatives methylundecylprodiginine and methyldodecylprodiginine (Figure 6).

### 3.3. Observation of Changes in Fatty Acid Composition Within Strains Under Heat Stress Conditions and with BCAAs Supplementation

**Figure 7:**
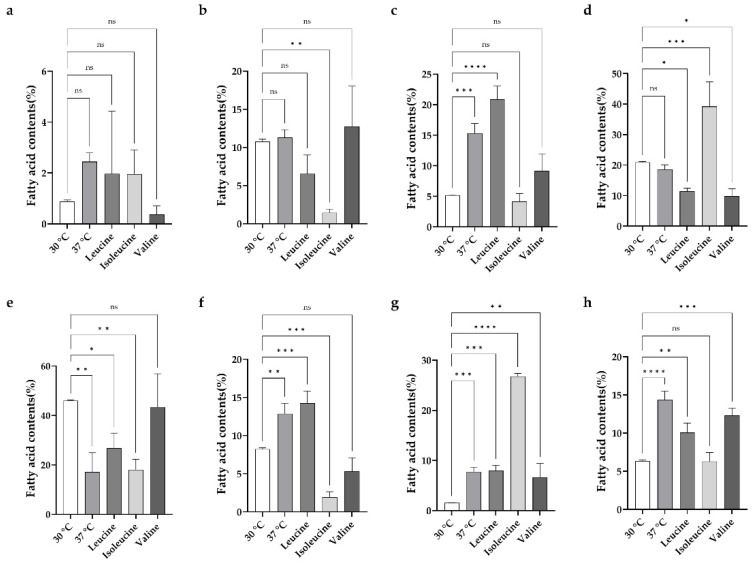
Fatty acid ratio of the M511 strains cultured under each condition. (**a**) 10-methyl-dodecanoic acid (aiC_13_), (**b**) 12-methyl-tridecanoic acid (iC_14_), (**c**) 13-methyl-tetradecanoic acid (iC_15_), (**d**) 12-methyl-tetradecanoic acid (aiC_15_), (**e**) 14-methyl-pentadecanoic acid (iC_16_), (**f**) 15-methyl-hexadecanoic acid (iC_17_), (**g**) 14-methyl-hexadecanoic acid (aiC_17_), and (**h**) hexadecanoic acid (palmitic acid) (C_16_). The figure indicates the change in fatty acid composition under each condition. The *p* values were calculated by comparing each treatment group with the 30 °C control. * *p* < 0.05; ** *p* < 0.01; *** *p* < 0.001; **** *p* < 0.0001; ns, not significant.

GC-MS analysis of the fatty acid profile extracted from the M511 strain revealed a total of seven BCFAs and one SCFA. At 30 °C, 14-methyl-pentadecanoic acid (iC_16_) dominated; at 37 °C, palmitic acid (C_16_) and minor BCFAs (iC_15_, aiC_17_) were more prominent. Upon BCAA supplementation, compared with the 37 °C no-supplementation group, isoleucine supplementation led to aiC_15_ and aiC_17_ becoming predominant, whereas iC_16_ predominated with valine. However, only a slight change in minor BCFAs was observed in the leucine supplementation group (Figure 7).

In the GC-MS chromatogram of the strain cultured at 30 °C, the peak with the highest intensity was 14-methyl-pentadecanoic acid (iC_16_), a BCFA with a methyl group attached to the 14th carbon atom, followed by 12-methyl-tetradecanoic acid (aiC_15_), 12-methyl-tridecanoic acid (iC_14_) in that order (Figure S1a). In contrast, significant differences in fatty acid composition were observed in the strain cultured under heat stress conditions at 37 °C. The GC-MS chromatogram was analyzed using Qualitative Analysis 10.0 software to calculate the relative ratios of each fatty acid. The results of the ratio analysis are presented in Table 1. As shown in Table 1, at 37 °C, the proportion of palmitic acid (C_16_) increased 2.25-fold (from 6.37% to 14.47%), whereas the proportion of BCFA decreased slightly (93.63% at 30 °C and 85.63% at 37 °C). Interestingly, the proportion of the significant even number BCFA, C_16_ (14-methyl-pentadecanoic acid), decreased 2.66-fold (from 45.97% at 30 °C to 17.24% at 37 °C), whereas aiC_15_ and iC_14_ remained relatively stable. In contrast, odd-number BCFAs, aiC_17_ (14-methyl-hexadecanoic acid), iC_15_ (13-methyl-tetradecanoic acid), and iC_17_ (15-methyl-hexadecanoic acid) increased 4.86, 2.95, and 1.56-fold, respectively. Subsequently, BCAAs were added to the growth medium of the bacteria grown at 37 °C to assess their impact on fatty acid metabolism. In these conditions, the proportion of total SCFA was slightly reduced, whereas that of total BCFA was slightly increased (1.1–1.5-fold in both cases), compared with the 37 °C culture group.

For instance, when leucine was added, it led to a slight reduction in total SCFA (1.56-fold) and a small enhancement of BCFAs (approximately 5%). Upon leucine addition, the relative ratios of aiC_13_, iC_14,_ and aiC_15_ were slightly decreased, whereas those of iC_15_, iC_16_, iC_17,_ and aiC_17_ were slightly increased, usually by less than 5% in both cases, compared to the 37 °C culture group.

Valine supplementation resulted in a slight reduction in the relative ratios of SCFA while modestly increasing the relative ratio of total BCFA. Upon valine addition, the relative ratios of aiC_13_, iC_15_, aiC_15_, iC_17,_ and aiC_17_ were slightly decreased, usually less than 2-fold except for aiC_13_, which was decreased 6.6-fold, whereas that of iC_16_ was clearly enhanced (2.5-fold) compared to the 37 °C culture group.

However, when isoleucine was added, the relative ratios of SCFA were reduced more than 2-fold, whereas the relative ratio of total BCFA was slightly enhanced compared with the 37 °C no supplementation group. Upon isoleucine addition, the relative ratios of iC_14_ (7.8-fold), iC_15_ (>3-fold), and iC_17_ (6.55-fold) were markedly reduced compared to the 37 °C no supplementation group. In contrast, the relative ratios of aiC_15_ (4.45-fold) and aiC_17_ (3.44-fold) increased, while aiC_13_ and iC_16_ remained unchanged. Statistical analyses of fatty acid profiles were performed as described in Supplementary Tables S7 and S8.

## 4. Discussion

In this study, we demonstrated that heat stress reduces 3.46-fold the biosynthesis of prodiginine, promotes the biosynthesis of branched-chain alkylprodiginine derivatives, and induces changes in fatty acid metabolism in the strain, resulting primarily in a 2.25-fold increase in the ratio of SCFAs in *S. coelicolor* M511 grown at 37 °C rather than at 30 °C. Additionally, we conducted experiments at 34 °C and 37 °C under the same conditions. Whereas distinct changes in prodiginine derivatives and fatty acids were not observed at 34 °C, a distinct pattern of change was detected at 37 °C.

The biosynthesis of branched-chain alkylprodiginine derivatives was previously reported in a study on the *S. coelicolor* (*ΔredP*) mutant strain, SJM1 [24]. RedP is the enzyme that initiates undecylprodiginine biosynthesis in *S. coelicolor* by condensing an acyl-CoA with malonyl-ACP/RedQ. RedP is homologous to FabH, which catalyzes the same reaction for initiation of fatty acid biosynthesis [24]. In the SJM1 strain lacking the *redP* gene, the total poridiginine production was substantially reduced, consistent with our 37 °C experiments. This suggested that RedP might be heat sensitive. The optimal temperature for RedP remains undetermined; however, previous reports have evaluated the activity of RedP and FabH under 30 °C conditions [23]. This putative heat sensitivity of RedP may account for the reduced production of undecylprodiginine. Under conditions of heat stress, RedP might functionally be compromised, and the FabH enzyme may catalyze the initial step of undecylprodiginine instead of RedP. Since *S. coelicolor* FabH is known to exhibit a higher preference for branched-chain substrates than for straight-chain substrates [15,16,33,34]. Accordingly, under heat-stress conditions, FabH may initiate prodiginine derivative biosynthesis using a different precursor than RedP [22], leading to the biosynthesis of the branched-chain alkylprodiginine derivatives detected. *S. coelicolor* utilizes the type II fatty acid synthase (FAS) pathway for fatty acid biosynthesis, with the initial condensation reaction catalyzed by the FabH enzyme [33,34]. The strain typically produces approximately 80–90% of its fatty acids as BCFAs, with SCFAs accounting for a relatively low proportion [15,16]. This is because the FabH of *S. coelicolor* exhibits a high substrate preference for branched-chain acyl-CoA [15]. On the other hand, *E. coli*, the first type II FAS to be characterized, produces only SCFAs by condensing acetyl-CoA and propionyl-CoA via ecFabH (FabH of *Escherichia coli*) [35]. These differences are attributed to the substrate specificity differences between FabH in the two species.

Furthermore, heat stress is known to induce changes in cellular membrane lipid composition in various bacteria [29,36,37,38]. SCFAs are known to enhance the structural stability of cell membranes, contributing to heat stress adaptation. A representative case, hexadecanoic acid (C_16_), is observed at high ratios in various bacteria. Due to its saturated, straight, long acyl-chain structure, it contributes to high melting points and thus enhances membrane stability [39,40]. In our study, the ratio of hexadecanoic acid increased by approximately 2.25-fold in the M511 strain cultured at 37 °C compared to 30 °C (Table 1). This result can be interpreted as maintaining its membrane stability under heat stress conditions. When BCAAs such as leucine, isoleucine, and valine were supplemented to the M511 strain under high-temperature stress conditions, the total proportion of BCFAs partially returned to the 30 °C level, and the relative abundance of specific BCFAs increased significantly. The catabolism of leucine, isoleucine, and valine yields isovaleryl-CoA, 2-methylbutyryl-CoA, and isobutyryl-CoA, respectively, through the oxidative decarboxylation reaction of branched-chain 2-keto acids. These precursors are subsequently converted into specific BCFAs such as iC_15_, iC_17_, aiC_15_, aiC_17_, iC_14_, and iC_16_ [41]. In this study, similar patterns of fatty acid composition changes were observed under high-temperature stress cultivation conditions, as shown in Table 1. The most significant changes were observed in the isoleucine-added group. The catabolism of isoleucine leads to the generation of 2-methylbutyryl-CoA, which serves as a precursor for the biosynthesis of BCFAs such as aiC_15_ and aiC_17_ by FabH.

Additionally, 2-methylbutyryl-CoA can serve as a substrate in the prodiginine biosynthesis pathway catalyzed by FabH, leading to the production of methyl-undecylprodiginine, a branched-chain derivative of undecylprodiginine. In this study, isoleucine is the BCAA exerting the greatest influence on precursor supply for fatty acid and undecylprodiginine biosynthesis under heat stress conditions. In this study, we demonstrated that heat stress–induced changes in fatty acid synthesis can alter undecylprodiginine production, highlighting a potential strategy to generate structurally modified derivatives of specialized metabolites. Additionally, this study presents a novel strategic approach that enables the production of various metabolite derivatives with differentiated or enhanced biological activity compared to the parent compound.

## Figures and Tables

**Table 1:** Fatty acid ratio of the M511 strains cultured under each condition.

Fatty Acid Type	% of Total Fatty Acid Pool in:				
	30 °C	37 °C	Leucine	Isoleucine	Valine
Total BCFAs	93.63	85.63	89.946	93.69	87.66
10-methyl-dodecanoic acid (aiC_13_)	0.89 ± 0.05	2.45 ± 0.34	1.97 ± 2.45	1.96 ± 0.94	0.37 ± 0.33
12-methyl-tridecanoic acid (iC_14_)	10.8 ± 0.3	11.33 ± 0.97	6.54 ± 2.47	1.45 ± 0.48	12.75 ± 5.29
13-methyl-tetradecanoic acid (iC_15_)	5.19 ± 0.73	15.34 ± 1.59	20.87 ± 2.18	4.15 ± 1.3	9.2 ± 2.77
12-methyl-tetradecanoic acid (aiC_15_)	20.94 ± 0.24	18.6 ± 1.54	11.41 ± 1.06	39.23 ± 8.03	9.9 ± 1.34
14-methyl-pentadecanoic acid (iC_16_)	45.97 ± 0.35	17.24 ± 7.74	26.88 ± 6.02	18.15 ± 4.22	43.45 ± 13.42
15-methyl-hexadecanoic acid (iC_17_)	8.24 ± 0.16	12.88 ± 1.36	14.26 ± 1.59	1.95 ± 0.71	6.67 ± 2.74
14-methyl-hexadecanoic acid (aiC_17_)	1.6 ± 0.04	7.79 ± 0.89	8.02 ± 1.05	26.8 ± 0.6	5.32 ± 1.78
Total SCFAs	6.37	14.37	10.054	6.31	12.34
Hexadecanoic acid (palmitic acid) (C_16_)	6.37 ± 0.93	14.37 ± 1.16	10.06 ± 1.27	6.3 ± 1.7	12.34 ± 0.96

BCFAS: branched-chain fatty acids; SCFAs: straight-chain fatty acids. *i-: iso-, ai-: anteiso-.*

## Data Availability

The original contributions presented in the study are included in the article; further inquiries can be directed to the corresponding authors.

## Supplementary Materials

The following supporting information can be downloaded at: https://www.mdpi.com/article/10.3390/microorganisms13112520/s1, Table S1: HPLC mobile phase gradient time table. Table S2: LC-MS mobile phase gradient time table. Table S3: Parameters for undecylprodiginine analysis on LC-MS. Table S4: Parameters for fatty acid analysis on GC-MS. Table S5: Statistical analysis of prodiginine in *Streptomyces coelicolor* M511 under different temperature and amino acid supplementation conditions. Table S6: Dunnett’s multiple comparisons test of prodiginine in *Streptomyces coelicolor* M511 under different temperature and amino acid supplementation conditions. Table S7: Statistical analysis of fatty acid profiles in *Streptomyces coelicolor* M511 under different temperature and amino acid supplementation conditions. Table S8: Dunnett’s multiple comparisons test of fatty acid profiles in *Streptomyces coelicolor* M511 under different temperature and amino acid supplementation conditions. Figure S1. GC-MS chromatogram of fatty acids from the M511 strain in each condition.
