# Supplementary material for: Effect of High-Temperature Stress on Fatty Acid Composition and Undecylprodiginine Biosynthesis in Streptomyces coelicolor M511
Source: Microorganisms. 2025 Nov 1;13(11):2520. doi: 10.3390/microorganisms13112520 (PMC12654105; doi:10.3390/microorganisms13112520)
Supplement: Supplementary file 1 [file microorganisms-13-02520-s001.zip › Supplementary Figure S1.pdf]

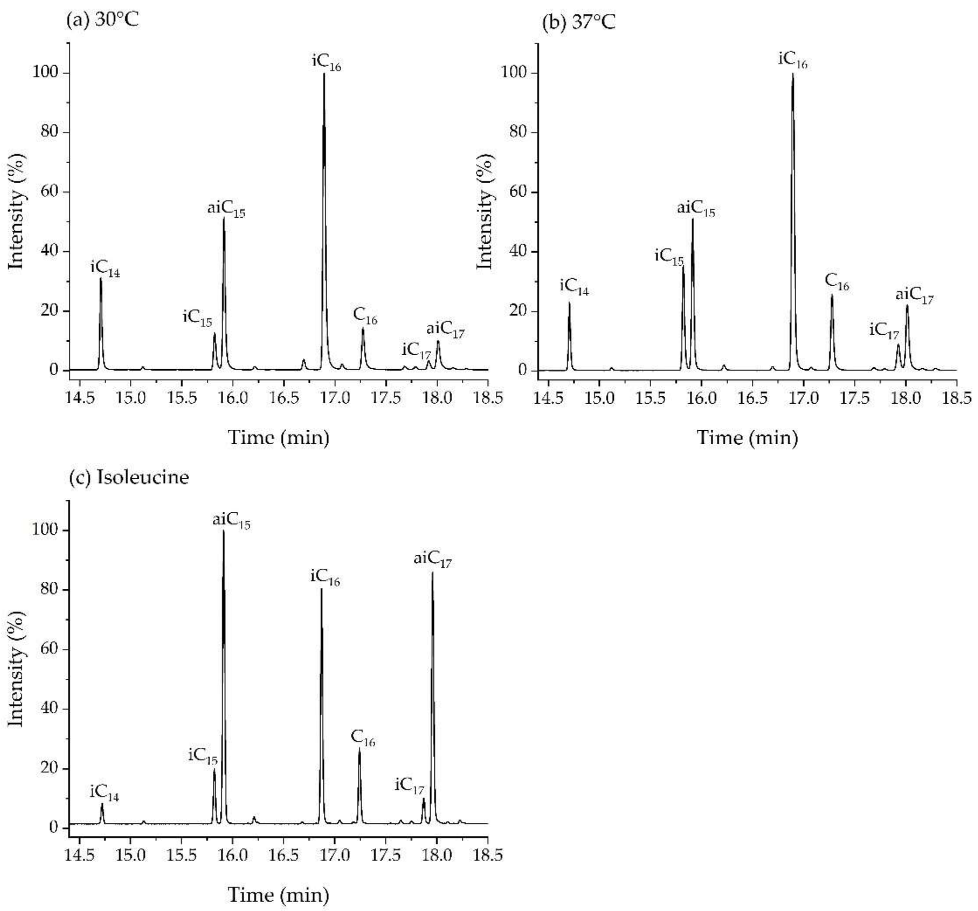

Supplementary Figure S1. GC-MS chromatogram of fatty acids from M511 strain in each condition.
i- : iso-type fatty acid
****ai-: anteiso-type fatty acid***
Fatty acids were extracted from cells cultured under each condition for GC-MS analysis. (a) 30 °C, (b) 37 °C, (c) 37 °C + isoleucine conditions, and the same fatty acid composition was confirmed in all three conditions.
